# Efficacy and Safety of Lenvatinib in Anaplastic Thyroid Carcinoma: A Meta-Analysis

**DOI:** 10.3389/fendo.2022.920857

**Published:** 2022-06-30

**Authors:** Dongmei Huang, Jinming Zhang, Xiangqian Zheng, Ming Gao

**Affiliations:** ^1^Department of Thyroid and Neck Tumor, Tianjin Medical University Cancer Institute and Hospital, National Clinical Research Center for Cancer, Key Laboratory of Cancer Prevention and Therapy, Tianjin's Clinical Research Center for Cancer, Tianjin, China; ^2^Department of Thyroid and Breast Surgery, Tianjin Union Medical Center, Tianjin, China; ^3^Tianjin Key Laboratory of General Surgery Inconstruction, Tianjin Union Medical Center, Tianjin, China

**Keywords:** anaplastic thyroid carcinoma, lenvatinib, efficacy, safety, meta-analysis

## Abstract

**Background:**

Lenvatinib has shown promising efficacy in targeted therapies that have been tested to treat anaplastic thyroid carcinoma (ATC) in both preclinical and clinical studies. The aim of this study was to evaluate the efficacy and safety of lenvatinib in the treatment of patients with ATC.

**Methods:**

PubMed, the Cochrane Library, Embase, and ClinicalTrials.gov were searched for potential eligible studies from inception to February 1, 2022. The outcomes included partial response (PR), stable disease (SD), disease control rate (DCR), median progression-free survival (mPFS), and median overall survival (mOS). Effect sizes for all pooled results were presented with 95% CIs with upper and lower limit.

**Results:**

Ten studies met the inclusion criteria. The aggregated results showed that the pooled PR, SD, and DCR were 15.0%, 42.0%, and 63.0%, respectively. The pooled mPFS and mOS were 3.16 (2.18–5.60) months and 3.16 (2.17–5.64) months, respectively. Furthermore, PFS rate at 3 months (PFSR-3m), PFSR-6m, PFSR-9m, PFSR-12m, and PFSR-15m were 52.0%, 22.5%, 13.9%, 8.4%, and 2.5%, respectively. Meanwhile, the 3-month OS rate (OSR-3m), OSR-6m, OSR-9m, OSR-12m, and OSR-15m were 64.0%, 39.3%, 29.7%, 18.9%, and 14.2%, respectively. The most common adverse events (AEs) of lenvatinib were hypertension (56.6%), proteinuria (32.6%), and fatigue (32%).

**Conclusions:**

This meta-analysis showed that lenvatinib has meaningful antitumor activity, but limited clinical efficacy in ATC.

**Systematic Review Registration:**

PROSPERO [https://www.crd.york.ac.uk/PROSPERO/], identifier [CRD42022308624].

## Introduction

Anaplastic thyroid carcinoma (ATC), a malignancy derived from undifferentiated thyroid follicular cells (1), accounts for 1%–2% of all thyroid cancers but has a poor prognosis, which accounts for 50% of all thyroid cancer-related deaths (2, 3). Most patients with ATC are older, often present with large, very rapidly growing tumors that often cause airway and esophagus compression, and even about half of them have distant metastatic disease at diagnosis. Among patients with ATC, the median survival time was 3–4 months and the 1-year survival rate was approximately 18%–20% (2, 4, 5). Up to now, there are no effective therapeutic options to treat ATC (6). Recently, in both preclinical and clinical studies, some novel targeted therapies have been tested for treating ATC, but had limited efficacy while lenvatinib has shown some promising and potential results (7, 8).

Lenvatinib is a multi-target antiangiogenetic broad-spectrum tyrosine kinase inhibitor (TKI) that can inhibit various signal receptors (VEGFR 1-3, FGFR 1-4, PDGFR-α, RET, and KIT proto-oncogenes) (9–12). In a global phase III study, lenvatinib showed a promising and meaningful efficacy in differentiated thyroid carcinoma (9). Recently, lenvatinib has been regarded as a promising target drug of ATC in Japan due to its significant antitumor effect (13). Evidence from the work of Iwasaki et al. (14) suggested that lenvatinib had a good disease control rate (DCR) and overall survival rate in patients with ATC. However, according to many different clinical studies, great differences in tumor response and survival in ATC patients treated with lenvatinib have been demonstrated. Therefore, this meta-analysis aimed to elucidate the efficacy and safety of lenvatinib in ATC, and hope to offer some guidance for clinical treatment of ATC.

## Methods

### Protocol and Registration

We have registered our protocol on PROSPERO (registration number: CRD42022308624). This meta-analysis followed the Preferred Reporting Items for Systematic Review and Meta-Analysis (PRISMA) statement (15). The PRISMA checklist is provided elsewhere (**Supplementary Table S1**).

### Search Strategy and Eligibility Criteria

PubMed, the Cochrane Library, Embase, and ClinicalTrials.gov were searched for potential eligible studies. The search was performed from inception to February 1, 2022. The search keywords were “thyroid carcinoma, anaplastic” and “lenvatinib” and the search strategy in PubMed was as follows: Thyroid Carcinoma, Anaplastic [Mesh] OR Anaplastic Thyroid Carcinoma [Title/Abstract] OR Anaplastic Thyroid Carcinomas [Title/Abstract] OR Carcinoma, Anaplastic Thyroid [Title/Abstract] OR Carcinomas, Anaplastic Thyroid [Title/Abstract] OR Thyroid Carcinomas, Anaplastic [Title/Abstract] OR Thyroid Cancer, Anaplastic [Title/Abstract] OR Anaplastic Thyroid Cancer [Title/Abstract] OR Anaplastic Thyroid Cancers [Title/Abstract] OR Cancer, Anaplastic Thyroid [Title/Abstract] OR Cancers, Anaplastic Thyroid [Title/Abstract] OR Thyroid Cancers, Anaplastic [Title/Abstract] AND Lenvatinib [Mesh] OR 4-(3-chloro-4-((cyclopropylaminocarbonyl)amino)phenoxy)-7-methoxy-6-quinolinecarboxamide [Title/Abstract] OR N-(4-((6-carbamoyl-7-methoxyquinolin-4-yl)oxy)-2-chlorophenyl)-N’-cyclopropylurea [Title/Abstract] OR 4-(3-chloro-4-(N’-cyclopropylureido)phenoxy)-7-methoxyquinoline-6-carboxamide [Title/Abstract] OR lenvatinib mesylate [Title/Abstract])) OR (E7080 mesylate [Title/Abstract] OR monomethanesulfonate [Title/Abstract] OR lenvatinib mesylate [Title/Abstract] OR lenvatinib methanesulfonate [Title/Abstract] OR Lenvima [Title/Abstract] OR E-7080 mesylate [Title/Abstract] OR E 7080 [Title/Abstract] OR 4-(3-chloro-4-(((cyclopropylamino)carbonyl)amino)phenoxy)-7-hydroxy-6-quinolinecarboxamide [Title/Abstract] OR E-7080 [Title/Abstract] OR ER-203492-00 [Title/Abstract] OR E7080 [Title/Abstract] OR lenvatinib metabolite M2 [Title/Abstract]. No language, region, ethnicity, age, or payment restrictions were imposed during the search process.

Inclusion criteria were as follows (1): studies including patients confirmed with ATC; (2) studies involving patients treated with lenvatinib; and (3) studies reporting either efficacy and/or safety endpoints. Exclusion criteria were as follows: (1) sample size less than 10 patients; and (2) article type: case report, review, conference abstract, and cell or animal study.

### Quality Assessment

Methodological index for non-randomized studies (MINORS) evaluates single-arm studies (16). JBI Critical Appraisal Checklist for Case Series evaluates retrospective studies without a comparison group (17).

### Data Extraction

Two investigators independently made study selection. If there were any differences between them, the third author would discuss with them together. Information on the following characteristics of included studies was recorded: authors, study type, sample size, age, criteria for tumor response [partial response (PR), stable disease (SD), and DCR], adverse events (AEs), and reported endpoints.

### Statistics

Analysis of pooled PR, SD, and DCR, and of the pooled K-M curves of ATC patients treated with lenvatinib was performed using R version 3.6.3. Effect sizes for all pooled results were presented with 95% CIs with upper and lower limit. Heterogeneity between studies was examined using the Cochrane Q chi-square test and *I*^2^ statistic. When *I*^2^ ≤ 50%, use the fixed-effects model; otherwise, use the random-effects model. For pooled results with high heterogeneity, the sensitivity analysis was performed by excluding each study individually. Begg’s test, Egger’s test, and the trim-and-fill method were used to assess publication bias. *p* < 0.05 was considered statistically significant.

## Results

### Search Results and Study Quality Assessment

**Figure 1:**
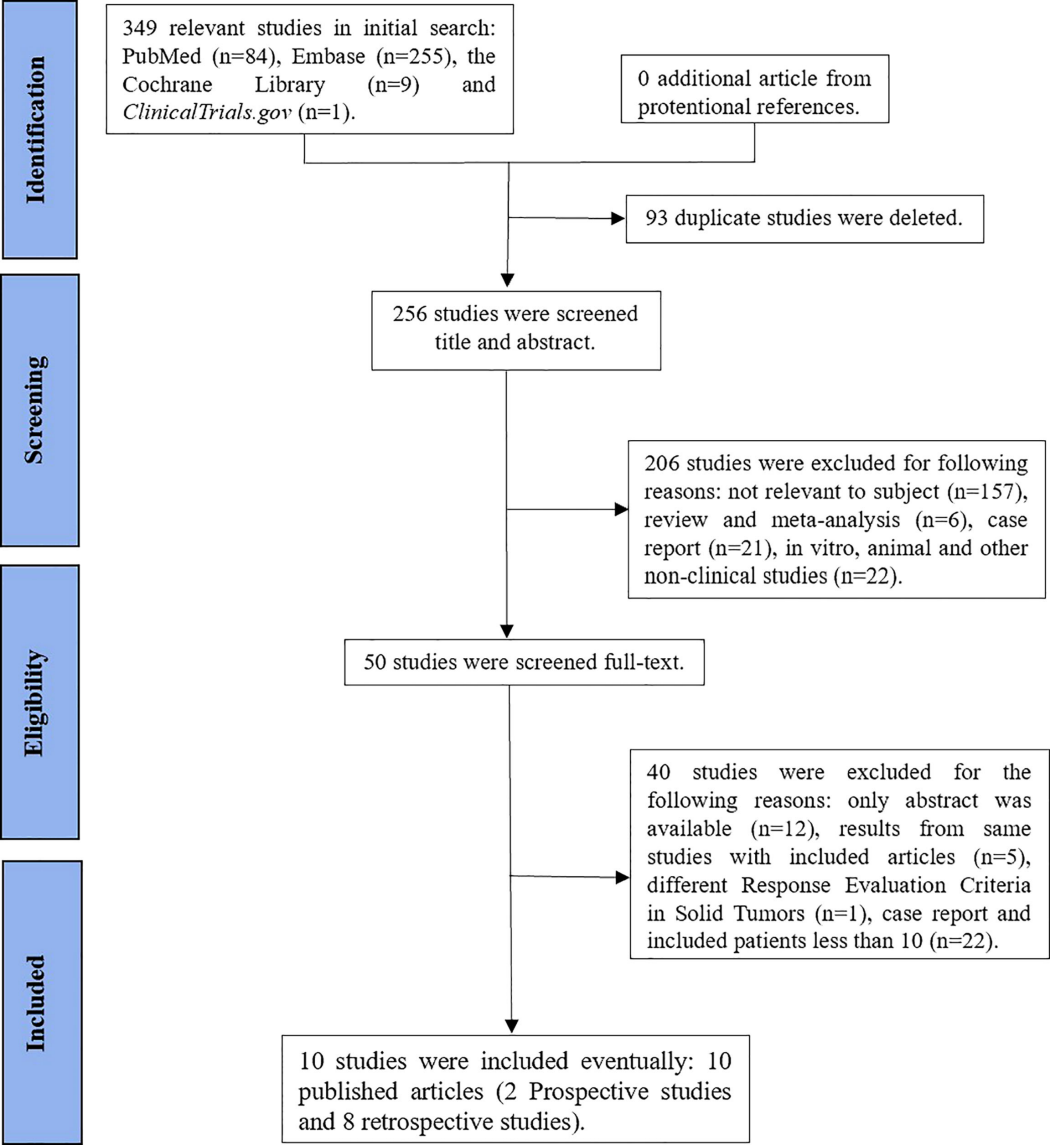
Flowchart of the studies’ selection process.

We initially identified 349 studies. Finally, our study included 10 studies, namely, 2 prospective studies (13, 18) and 8 retrospective studies (19–26) (**Figure 1**). The characteristics of the study are shown in **Table 1**.

**Table 1:** Characteristic of included studies.

Study	Country	Study type	Recruitment/case review period	Intervention	*n*	Median age (range)	M/F	Primary endpoints	Criteria for response	Criteria for AEs
Fukuda (2020) (19)	Japan	Retrospective study	2012–2019	Lenvatinib	13	68 (39–80)	4/9	PFS, OS, PR, SD, DCR	RECIST	AEs were not reported
Ishihara (2021) (20)	Japan	Retrospective study	2014–2019	Lenvatinib	10	69 (40–77)	3/7	OS, PR, SD, AEs	RECIST	CTCAE
Iwasaki (2021) (26)	Japan	Retrospective study	2015–2020	Lenvatinib	32	77 (42–89)	14/18	AEs, PR, SD, OS	RECIST	CTCAE
Iyer (2018) (21)	America	Retrospective study	2015–2016	Lenvatinib	10	NR	NR	PFS, OS, PR, SD, AEs	RECIST	CTCAE
Kim (2020) (22)	Korea	Retrospective cohort study	2016–2019	Lenvatinib	14	65.6 (59.7–72.1)	5/9	PFS, OS, PR, SD, DCR, AEs	RECIST	CTCAE
Park (2021) (23)	Korea	Retrospective study	1995–2020	Lenvatinib	11	NR	NR	PR, SD,	RECIST	AEs were not reported
Sparano (2021) (24)	French	Retrospective review	2015–2019	Lenvatinib	15	67.1 ± 7.6	9/6	PFS, OS, PR, SD	RECIST	CTCAE
Takahashi (2019) (13)	Japan	A non-randomized, open-label, multicenter, Phase II study	2012–2015	Lenvatinib	17	65 (36–84)	6/11	AEs, PFS, OS, PR, SD, DCR	RECIST	CTCAE
Wirth (2021) (18)	3 countries	An open-label, multicenter, international, phase II study	NR	Lenvatinib	34	NR	13/21	PFS, OS, DCR, PR, SD, AEs	RECIST	CTCAE
Yamazaki (2021) (25)	Japan	Retrospective study	2015–2019	Lenvatinib	20	73.6 ± 9.0	9/11	OS, PR	RECIST	AEs were not reported

Two single-arm studies (13, 18) scored 12 points using the MINORS index, which were acceptable for the current meta-analysis. Eight retrospective studies (19–26) were evaluated using the JBI Critical Appraisal Checklist for Case Series (**Table 2**).

**Table 2:** Quality assessment of included studies.

Study												
**A. MINORS index for included non-randomized studies**												
Study	I		II	III		IV		V	VI	VII	VIII	Total
Takahashi et al. (2019) (13)	2		2	2		2		0	2	2	0	12
Wirth et al. (2021) (18)	2		1	2		2		0	1	2	2	12
**B. JBI critical appraisal checklist for case series for included retrospective studies**												
Study	Q1	Q2	Q3		Q4	Q5	Q6	Q7	Q8	Q9	Q10	Overall appraisal
Fukuda et al. (2020) (19)	Yes	Yes	Yes		Yes	Yes	Yes	Yes	Yes	Yes	Yes	Include
Ishihara et al. (2021) (20)	Yes	Yes	Yes		Yes	Yes	Yes	Yes	Yes	Yes	Yes	Include
Iwasaki et al. (2021) (26)	Yes	Yes	Yes		Yes	Yes	Yes	Unclear	Yes	Yes	Yes	Include
Iyer et al. (2018) (21)	Yes	Yes	Yes		Yes	Yes	Yes	Yes	Yes	Yes	Yes	Include
Kim et al. (2021) (22)	Yes	Yes	Yes		Yes	Yes	Yes	Yes	Yes	Yes	Yes	Include
Park et al. (2021) (23)	Yes	Yes	Yes		Yes	Yes	Yes	Yes	Yes	Yes	Yes	Include
Sparano et al. (2021) (24)	Yes	Yes	Yes		Yes	Yes	Yes	Yes	Yes	Yes	Yes	Include
Yamazaki et al. (2021) (25)	Yes	Yes	Yes		Yes	Yes	Yes	Unclear	Yes	Yes	Yes	Include

### Efficacy

#### Tumor Response

**Figure 2:**
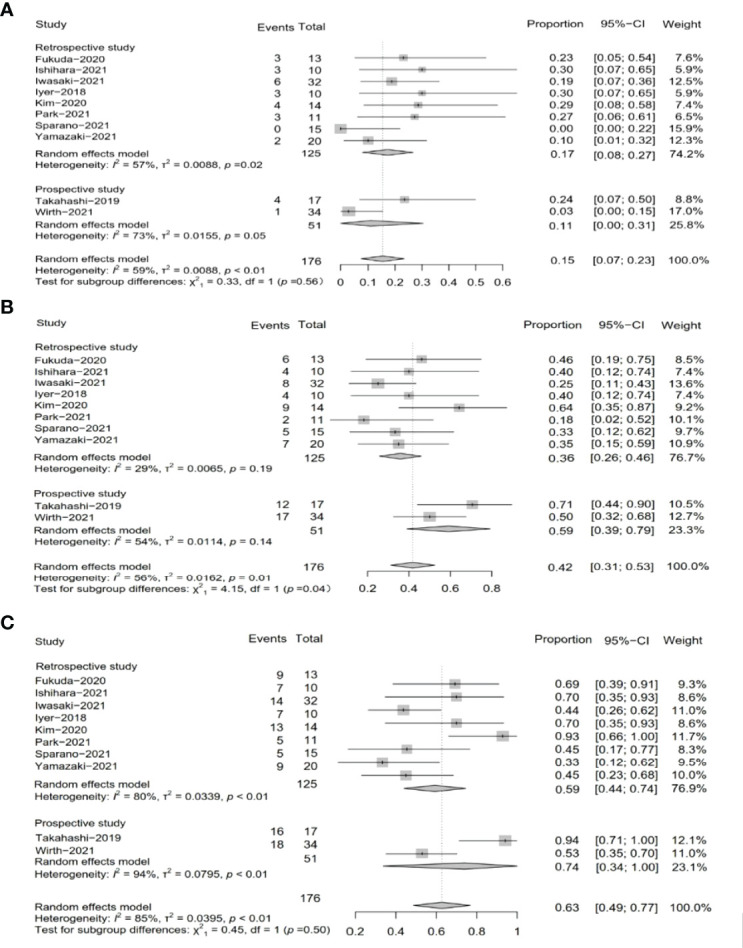
Pooled results of tumor response by study type subgroup. **(A)** Pooled results of PR in total by research type subgroup. **(B)** Pooled results of SD in total by research type subgroup. **(C)** Pooled results of DCR in total by research type subgroup.

We extracted efficacy measures from each study which included in this meta-analysis (**Table 3**). These studies were divided into two subgroups, namely, the subgroup of retrospective studies and the subgroup of prospective studies according to study types. Nine studies reported PR as an outcome of clinical activity. The pooled PR was 15.0% (95% CI, 7%–23%, *I*^2^ = 59.0%, *p* < 0.01), and the pooled PR in subgroups was different (**Figure 2A**). In the subgroups of the retrospective study, the pooled PR was 17% (95% CI, 8%–27%, *I*^2^ = 57%, *p* = 0.02), while the other subgroups showed a pooled PR of 11% (95% CI, 0%–31%, *I*^2^ = 73%, *p* = 0.05).

**Table 3:** Efficacy measurement in each study.

Study	No.	PR, *N* (%)	SD, *N* (%)	PD, *N* (%)	NE, *N* (%)	ORR, *N* (%)	DCR, *N* (%)	Median PFS (m)	Median OS (m)
Fukuda (2020) (19)	13	3 (23)	6 (46.2)	4 (30.8)	–	3 (23)	9 (69.2)	3.8 (1.8–6.4)	10.2 (3.7–17.6)
Ishihara (2021) (20)	10	3 (30)	4 (40)	2 (20)	1 (10)	3 (30)	7 (70)	–	4.75 (1.9–13.1)
Iwasaki (2021) (26)	32	6 (18.8)	8 (25)	12 (37.5)	6 (18.8)	6 (18.8)	14 (43.8)	–	3.2 (0.5–28.9)
Iyer (2018) (21)	10	3 (30)	4 (40)	1 (10)	–	3 (30)	7 (70)	2.6 (1.8–NR)	3.9 (2.5–NR)
Kim (2020) (22)	14	4 (28.6)	9 (64.2)	1 (7.1)	–	4 (28.6)	13 (92.9)	5.7 (2.2–8.3)	6.7 (3.0–8.4)
Park (2021) (23)	11	3 (27.3)	2 (18.2)	6 (54.5)	–	3 (27.3)	5 (45.5)	–	–
Sparano (2021) (24)	15	0 (0)	5 (33.3)	6 (40)	–	0 (0)	5 (33.3)	–	2.7 (1.5–3.8)
Takahashi (2019) (13)	17	4 (23.5)	12 (70.6)	1 (5.9)	0 (0.0)	4 (23.5)	16 (94.1)	7.4 (1.7–12.9)	10.6 (3.8–19.8)
Wirth (2021) (18)	34	1 (2.9)	17 (50)	9 (26.5)	7 (20.6)	1 (2.9)	18 (52.9)	2.6 (1.4–2.8)	3.2 (2.8–8.2)
Yamazaki (2021) (25)	20	2 (10)	7 (35)	7 (35)	4 (35)	2 (10)	9 (45)	–	–

SD was reported in ten studies, which was 42% after being pooled (95% CI, 31%–53%, *I*^2^ = 56%, *p* = 0.01), while the subgroup of the retrospective study showed a pooled SD of 36% (95% CI, 26%–46%, *I*^2^ = 29%, *p* = 0.19), and the subgroup of the prospective study resulted in a pooled SD of 59% (95% CI, 39%–79%, *I*^2^ = 54%, *p* = 0.14) (**Figure 2B**).

Two subgroups of prospective studies and retrospective studies reported that the pooled DCR was 74% (95% CI, 34%–100%, *I*^2^ = 94%, *p* < 0.01) and 59% (95% CI, 44%–74%, *I*^2^ = 80%, *p* < 0.01), respectively. The total pooled DCR was 63% (95% CI, 49%–77%, *I*^2^ = 85%, *p* < 0.01) (**Figure 2C**).

#### Survival

**Figure 3:**
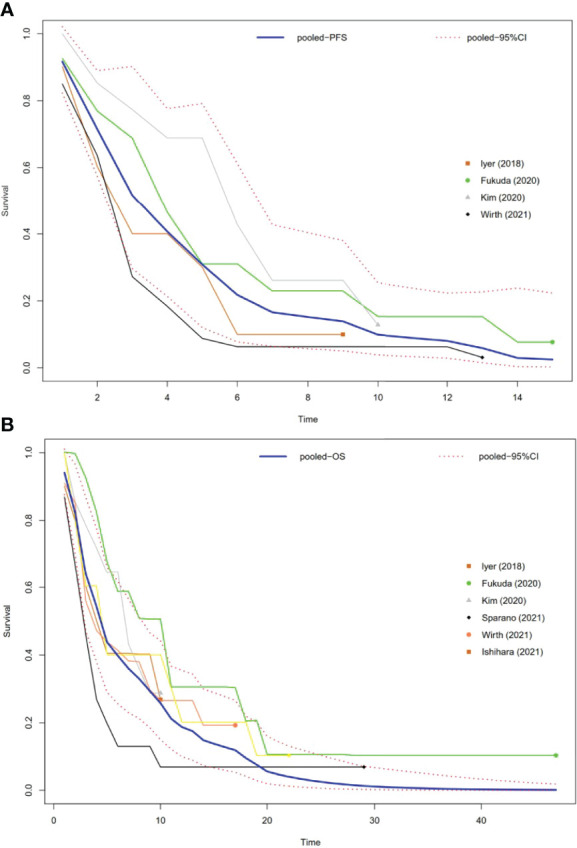
Pooled Kaplan–Meier survival curves of ATC patients. **(A)** Pooled Kaplan–Meier PFS curves of ATC patients. **(B)** Pooled Kaplan–Meier OS curves of ATC patients.

**Figure 4:**
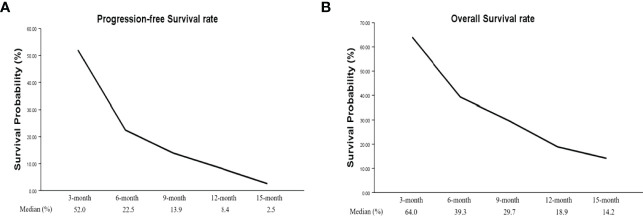
Tendency chart of pooled PFS rate (3–15 months, **A**) and pooled OS rate (3–15 months, **B**).

Four studies had PFS K-M curves (18, 19, 21, 22), and the pooled median progression-free survival (mPFS) was 3.16 (95% CI, 2.18–5.60) months (**Figure 3A**), with the PFS rate at 3 months (PFSR-3m), PFSR-6m, PFSR-9m, PFSR-12m, and PFSR-15m being 52.0%, 22.5%, 13.9%, 8.4%, and 2.5% (**Figure 4A**), respectively.

The OS K-M curves were reported in six studies (18–22, 24), and the pooled median overall survival (mOS) was 3.16 (95% CI, 2.17–5.64) months (**Figure 3B**), with the 3-month OS rate (OSR-3m), OSR-6m, OSR-9m, OSR-12m, and OSR-15m being 64.0%, 39.3%, 29.7%, 18.9%, and 14.2% (**Figure 4B**), respectively.

#### Safety—Adverse Events

Six studies reported AEs (13, 18, 21, 22, 24, 26). AEs were experienced by all patients, and most were manageable with dose adjustment and drug therapy. The most common AEs of lenvatinib in ATC were hypertension (56.6%), proteinuria (32.6%), and fatigue (32%) (**Table 4**).

**Table 4:** Safety measurements in each study.

Study	Hypertension	Proteinuria	Fatigue	Asthenia	Anorexia	Hypothyroidism	Stomatitis	Vomit	Thrombocytopenia
Iwasaki	24 (75%)	13 (40.6%)	4 (12.5%)	4 (12.5%)	15 (46.9%)	–	3 (9.4%)	1 (3.1%)	–
Iyer	7 (44%)	3 (19%)	7 (44%)	7 (70%)	–	4 (40%)	5 (50%)	2 (20%)	2 (20%)
Kim	12 (86%)	11 (79%)	11 (79%)	11 (78.6%)	12 (85.7%)	11 (78.6%)	3 (21%)	–	2 (14.3%)
Sparano	10 (43.5%)	–	10 (43.5%)	10 (66.7%)	5 (21.7%)	2 (13.3%)	–	1 (6.7%)	3 (20%)
Takahashi	14 (82%)	10 (59%)	10 (59%)	10 (58.8%)	14 (82%)	2 (11.8%)	8 (47%)	6 (35.3%)	5 (29.4%)
Wirth	8 (24%)	2 (6%)	3 (9%)	3 (8.8%)	–	–	–	1 (2.9%)	–

#### Publication Bias

Egger’s test, Begg’s test, and the trim-and-fill method were used to identify publication bias in the study. Pooled SD showed no significant publication bias in the included studies, *p* = 0.509 by Egger’s test and *p* = 0.588 by Begg’s test. Graphically, the funnel plot shows potential publication bias (Egger’s test, *p* < 0.05) on the estimated pooled PR and DCR (**Supplementary Figure S2**).

## Discussion

As a rare and lethal type of thyroid carcinoma, ATC has a poor prognosis, which reports that nearly 50% of patients had metastatic disease at diagnosis (27). Currently, there are limited options for treating ATC, with an estimated first-year mortality rate of 90% (3, 28). As previously reported, chemotherapies such as doxorubicin, paclitaxel, and cisplatin did not prolong survival in patients with ATC (29, 30). However, the results of Viglietto et al. showed that VEGF was overexpressed in ATC tissues and pointed out that VEGFR expression was also increased in the microvascular endothelial cells of ATC tumor specimens (31). Moreover, Haruhiko et al. proposed that FGFR4 was strongly expressed in ATC (32, 33), which suggested that ATC has many biological targets that can be inhibited and blocked by TKIs. Among these TKIs, some clinical data showed that lenvatinib might provide efficacious benefits to ATC patients (7, 13). To evaluate the efficacy and safety of lenvatinib in ATC patients, the data on tumor response, survival, and safety were extracted and analyzed in this meta-analysis.

Among all the studies, there were two single-arm, phase II studies, with a relatively large sample size, which may provide more reliable lines of evidence on the efficacy and safety of lenvatinib in ATC. One was a nonrandomized, open-label, multicenter, phase II study (13) including 17 patients, which demonstrated that the PR, SD, DCR, the mPFS, and the mOS were 24%, 71%, 94%, 7.4 months, and 10.6 months, respectively. The other single-arm, phase II study (18) on 34 patients showed that the PR, SD, DCR, mPFS, and mOS were 3%, 50%, 53%, 2.6 months, and 3.2 months, respectively. Differences between two prospective studies may be due to the different ethnicity, tumor pathology, or prior treatment. Our meta-analysis showed that pooled PR, pooled SD, and pooled DCR were 15%, 42%, and 63%, respectively, which demonstrated that lenvatinib showed a potential and meaningful antitumor activity in ATC patients. A study by Tahara et al. showed that 24% of ATC patients treated with lenvatinib achieved PR and 47% achieved SD (7), which was in accordance with the results of Koyama’s study (8) that reported 24% achieved PR after lenvatinib in 17 ATC patients. A study on 23 patients reported a DCR of 43.5% (14), and another study on ten patients showed a DCR of 70%, with an mPFS of only 2.7 months (21). In addition, it is questionable whether lenvatinib administration prolongs survival in ATC patients. In the analysis of survival data, the results showed that the pooled mOS and pooled mPFS were 3.16 months and 3.16 months, respectively, which indicated that lenvatinib has a limited efficacy in the treatment of ATC. It should be noted that a report on 124 patients, which was excluded from our study because of its criteria for response, showed a median OS of 101 days, which was in accordance with the results of our study (34), whereas Tahara et al. (7) reported that mPFS (7.4 months) and mOS (10.6 months) were longer with lenvatinib for the treatment of ATC. Therefore, we were unable to show a significant effect of lenvatinib in ATC on prolonging survival, which was also not demonstrated in previous studies (14, 21). However, compared with other multikinase inhibitors of VEGF receptors, such as pazopanib and sorafenib, which were used as monotherapy for ATC (35, 36), lenvatinib actually showed a meaningful antitumor activity in patients with ATC.

Medication safety is the focus of treatment. This meta-analysis showed that all patients experienced AEs and the most common AEs in ATC with lenvatinib were hypertension, proteinuria, fatigue, and asthenia, which are related toxic side effects of VEGF-targeted therapy (37). Hypertension was the most common AE and was well controlled by adjusting the dose and administering antihypertensive drugs. With regard to proteinuria, renal failure can be prevented by dose reduction and adequate withdrawal of lenvatinib (38). Lenvatinib-induced fatigue and asthenia can be improved with drug pauses and dose reduction. Furthermore, there were 3 patients who experienced severe hemoptysis and 2 patients underwent pneumothorax-related AEs, leading to death in our meta-analysis, which is unclear if lenvatinib was related. Lesions close to large vessels are at risk of bleeding and require careful administration (39). In particular, lesions with a history of external irradiation (40) or fistulae formed in the digestive tract or skin are at risk of rupture of the vessel wall (41). Although a rare complication, pneumothorax onset during lenvatinib treatment for thyroid carcinoma has already been described to be fatal (42). Therefore, careful management and continuous monitoring are required to avoid these AEs, which is critical to improving patient prognosis.

The study had some limitations. First, this meta-analysis had a strong heterogeneity among included studies, which may be caused by patient and tumor characteristics, such as tumor burden, prior treatment, and ethnicity. Second, although we included nearly all recent studies, only 10 eligible studies were included in our meta-analysis. Finally, most clinical research reports currently available are retrospective or single-arm studies with small sample sizes. Therefore, randomized and prospective studies with a large sample size are needed to evaluate the efficacy of lenvatinib in ATC.

## Conclusion

This study was the first systematic review of the efficacy and safety of lenvatinib in ATC. This meta-analysis showed that lenvatinib has a meaningful but limited clinical efficacy in ATC. Although most AEs can be controlled with dose adjustment or drug discontinuation, evaluation and prevention of fatal AEs are required during treatment. Studies with large sample sizes and randomized controlled trials are needed to confirm the efficacy and safety of lenvatinib in ATC, and provide stronger and high-quality evidence.

## Data Availability Statement

The original contributions presented in the study are included in the article/**Supplementary Material**. Further inquiries can be directed to the corresponding authors.

## Author Contributions

DH conceptualized and designed the study. DH and JZ critically assessed studies and extracted data. XZ and MG performed the analysis. DH and JZ wrote the manuscript. All authors contributed to the article and approved the submitted version.

## Funding

This work was supported by grants from the National Natural Science Foundation of China (81872169, 82172821, 82103386), Tianjin Municipal Science and Technology Project (19JCYBJC27400, 21JCZDJC00360) and Beijing-Tianjin-Hebei Basic Research Cooperation Project (20JCZXJC00120), The Science & Technology Development Fund of Tianjin Education Commission for Higher Education (2021ZD033), Tianjin Medical Key Discipline (Specialty) Construction Project.

## Conflict of Interest

The authors declare that the research was conducted in the absence of any commercial or financial relationships that could be construed as a potential conflict of interest.

## Publisher’s Note

All claims expressed in this article are solely those of the authors and do not necessarily represent those of their affiliated organizations, or those of the publisher, the editors and the reviewers. Any product that may be evaluated in this article, or claim that may be made by its manufacturer, is not guaranteed or endorsed by the publisher.

## Abbreviations

ATC, anaplastic thyroid carcinoma; PR, partial response; SD, stable disease; DCR, disease control rate; mPFS, median progression-free survival; mOS, median overall survival; VEGFR, vascular endothelial growth factor receptor; FGFR, fibroblast growth factor receptor; PDGFR, platelet-derived growth factor receptor; AEs, adverse events; MINORS, methodological index for non-randomized studies; CR, complete response; PD, progressive disease; ORR, overall response rate; NE, not estimable; RECIST, Response Evaluation Criteria in Solid Tumors; CTCAE, Common Terminology Criteria for Adverse Events; CBR, clinical benefit rate; NR, not estimable; PFSR, progression-free survival rate; OSR, overall survival rate; TKIs, tyrosine kinase inhibitors.
