# Supplementary material for: Efficacy and Safety of Lenvatinib in Anaplastic Thyroid Carcinoma: A Meta-Analysis
Source: Front Endocrinol (Lausanne). 2022 Jun 30;13:920857. doi: 10.3389/fendo.2022.920857 (PMC9279913; doi:10.3389/fendo.2022.920857)
Supplement: Supplementary file 2 [file DataSheet_2.docx]

Supplementary material S2

Supplemental 1: Sensitivity analysis for PR, SD and DCR


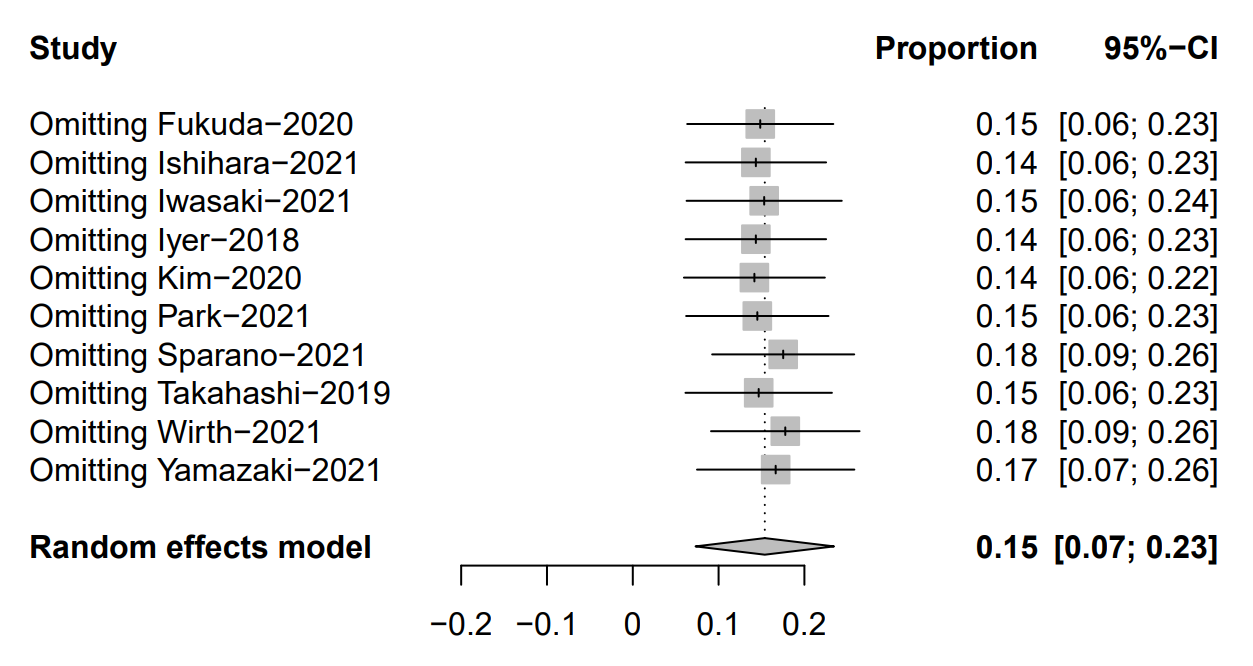


Figure S1. Sensitivity analysis for PR in ATC


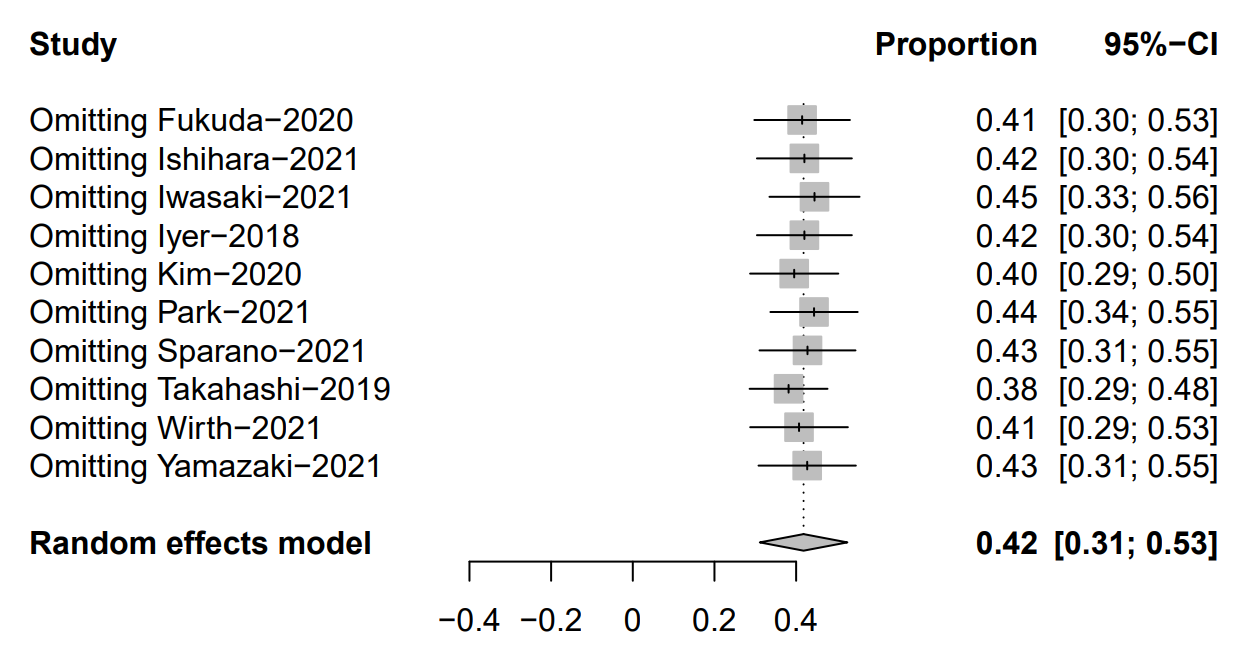


Figure S2. Sensitivity analysis for SD in ATC


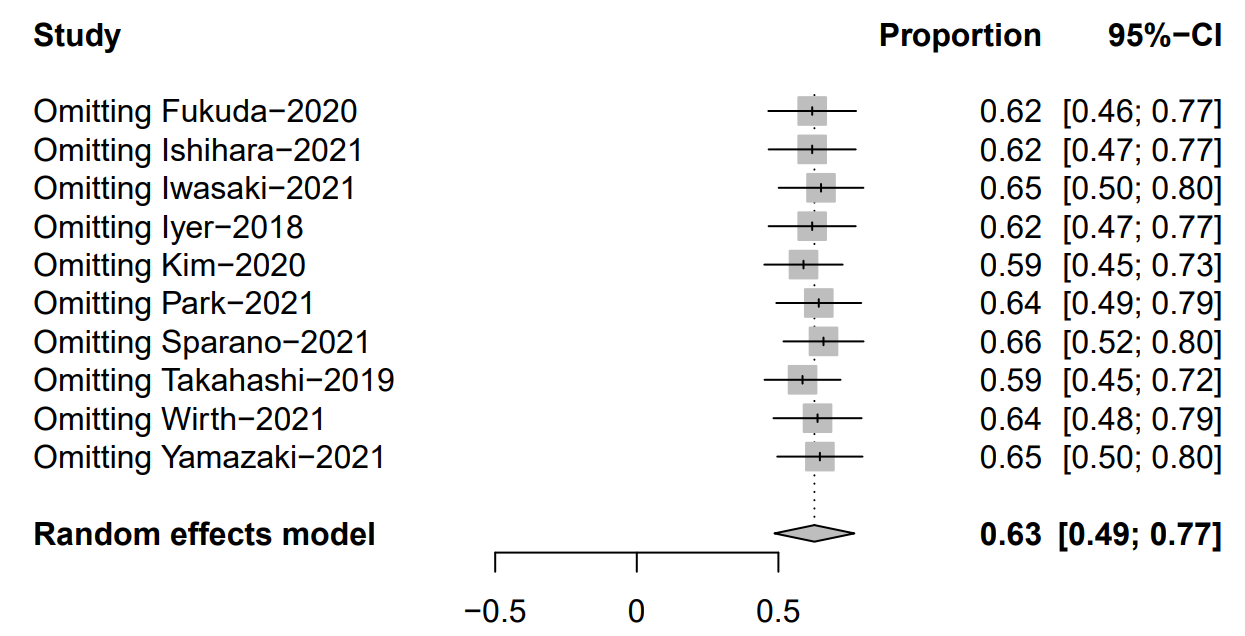


Figure S3. Sensitivity analysis for DCR in ATC

Supplemental 2: Publication biases for PR, SD and DCR


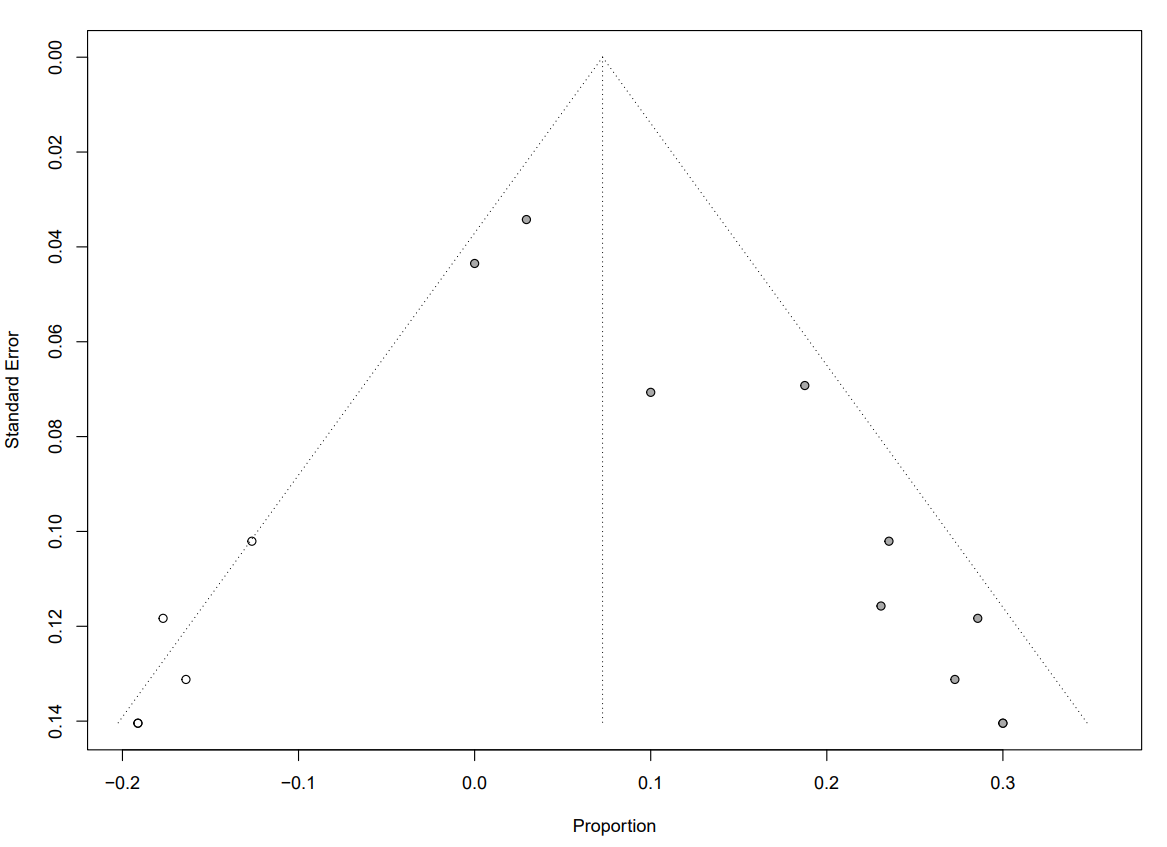


**Panel A: Funnel plot**

**Panel B: Trim-and-fill method**


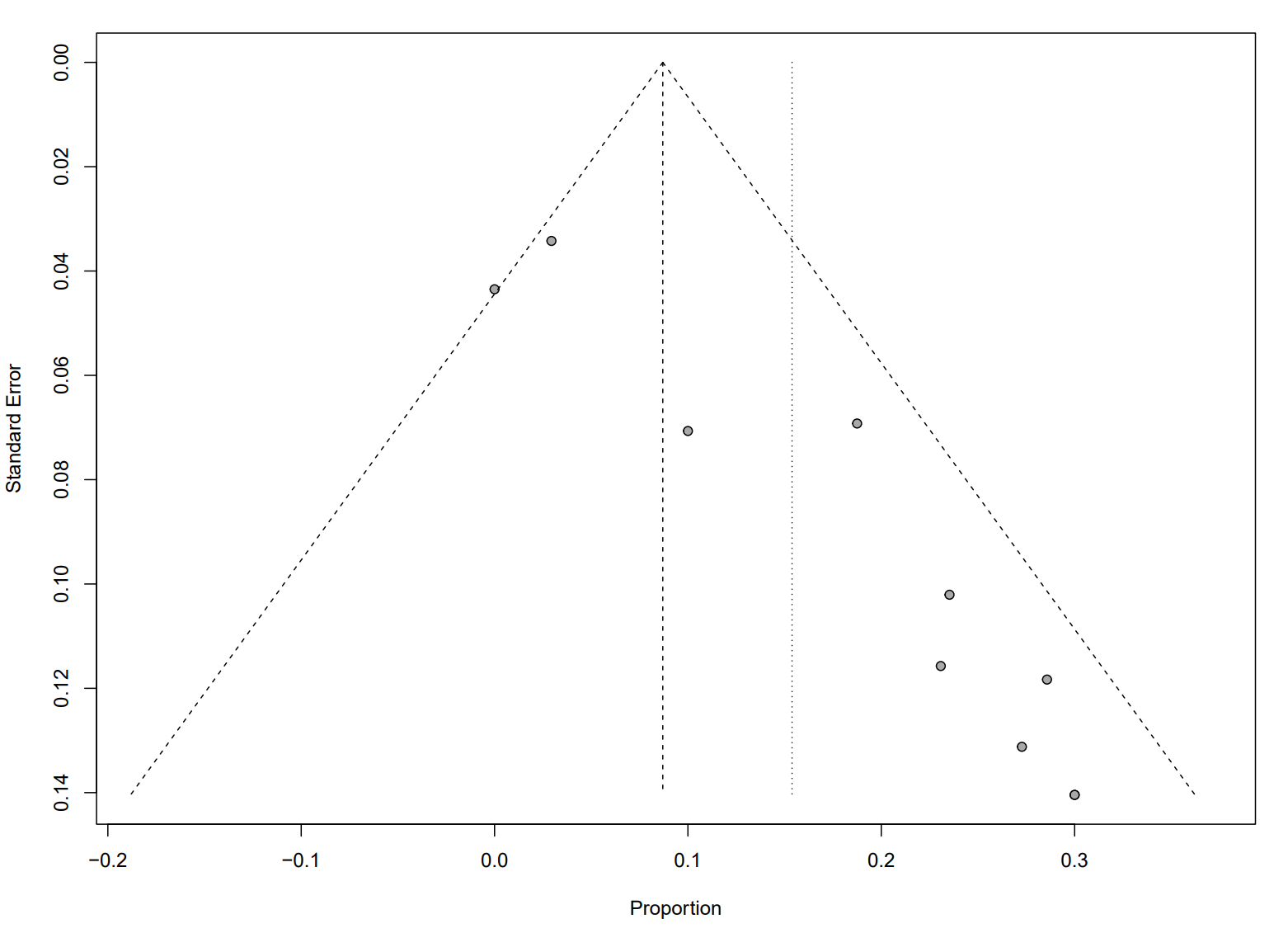


P value for Egger: 0.0245

P value for Begg: 0.0305

Figure S1. Funnel plot and trim-and-fill method examining the pooled PR in ATC.

**Panel A: Funnel plot**

**Panel B: Trim-and-fill method**

P value for Egger: 0.5090

P value for Begg: 0.5885


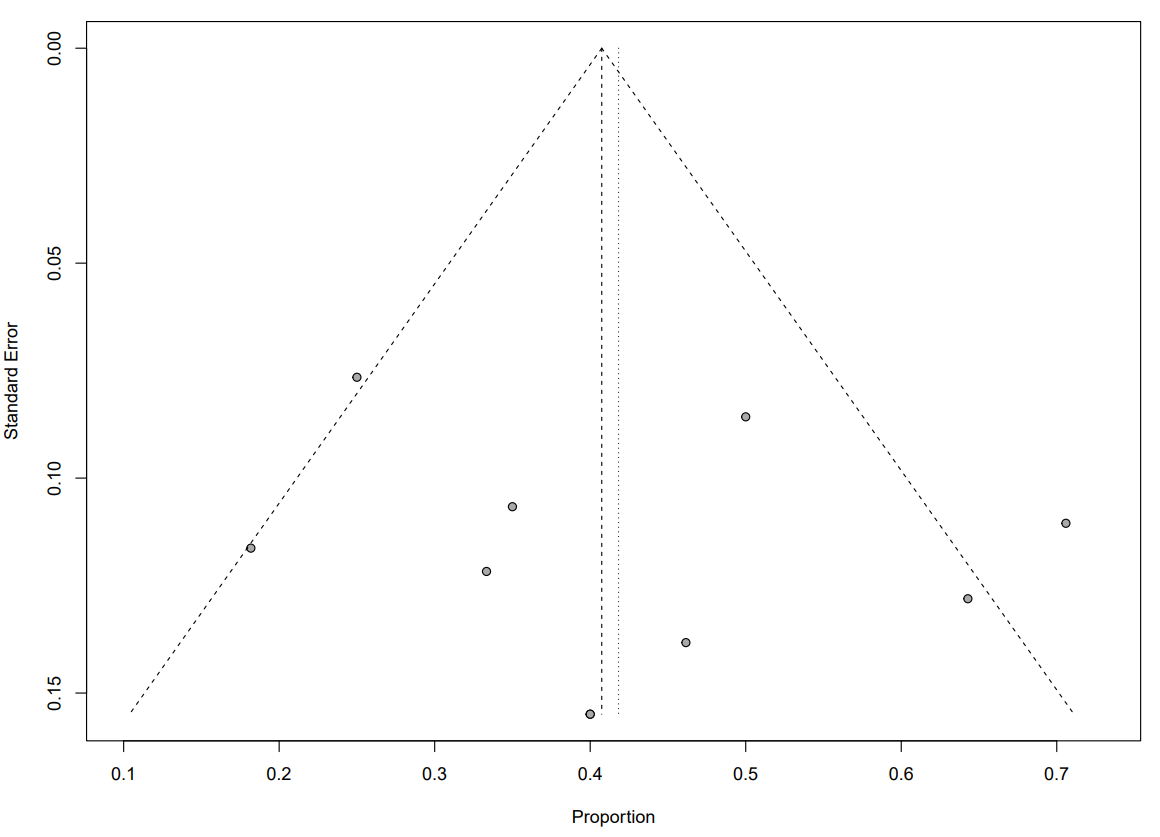

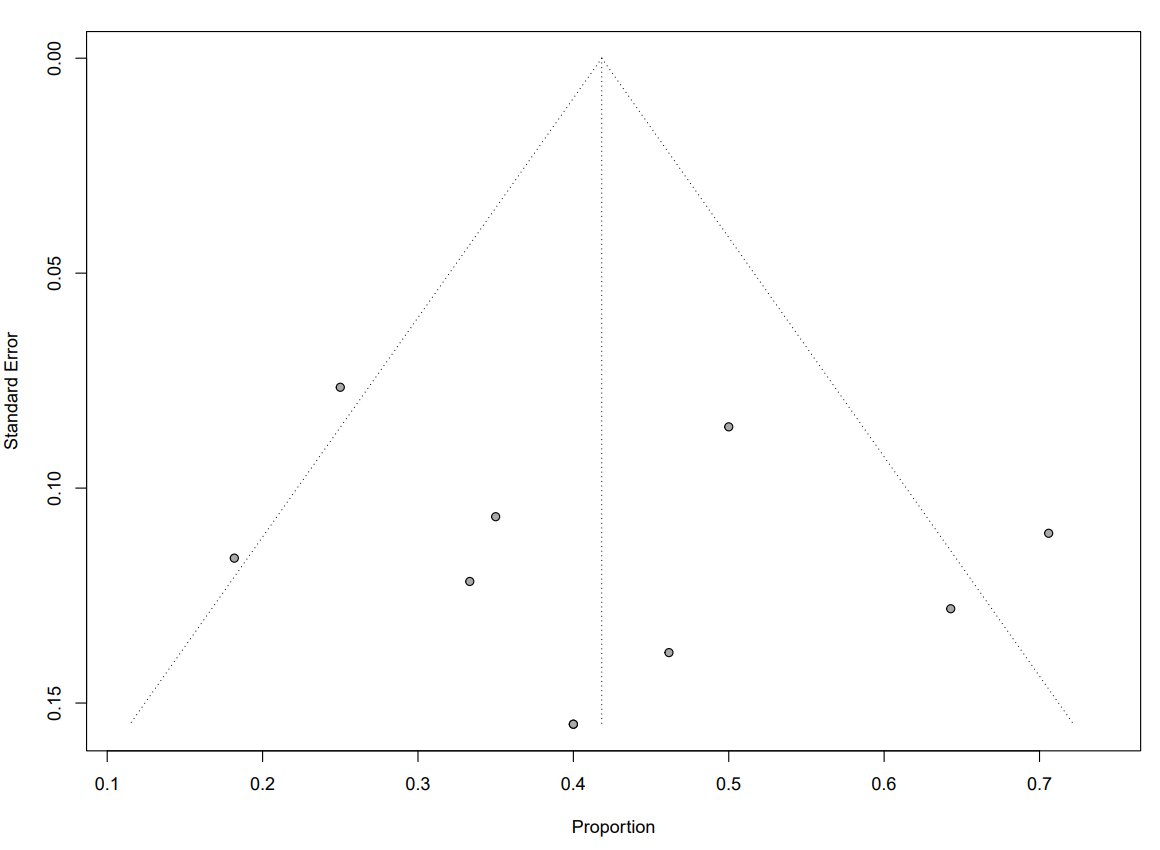


Figure S2. Funnel plot and trim-and-fill method examining the pooled SD in ATC.

**Panel A: Funnel plot**

**Panel B: Trim-and-fill method**

P value for Egger: 0.0413

P value for Begg: 0.7184


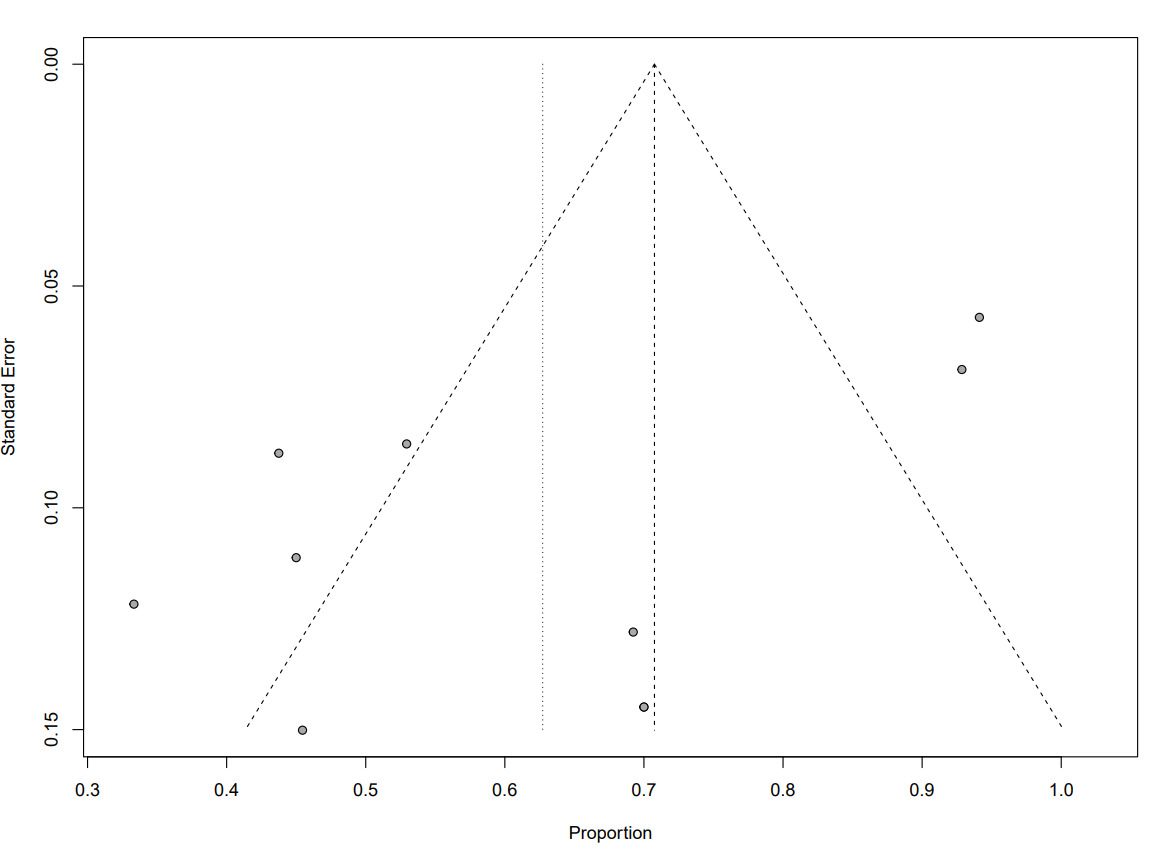

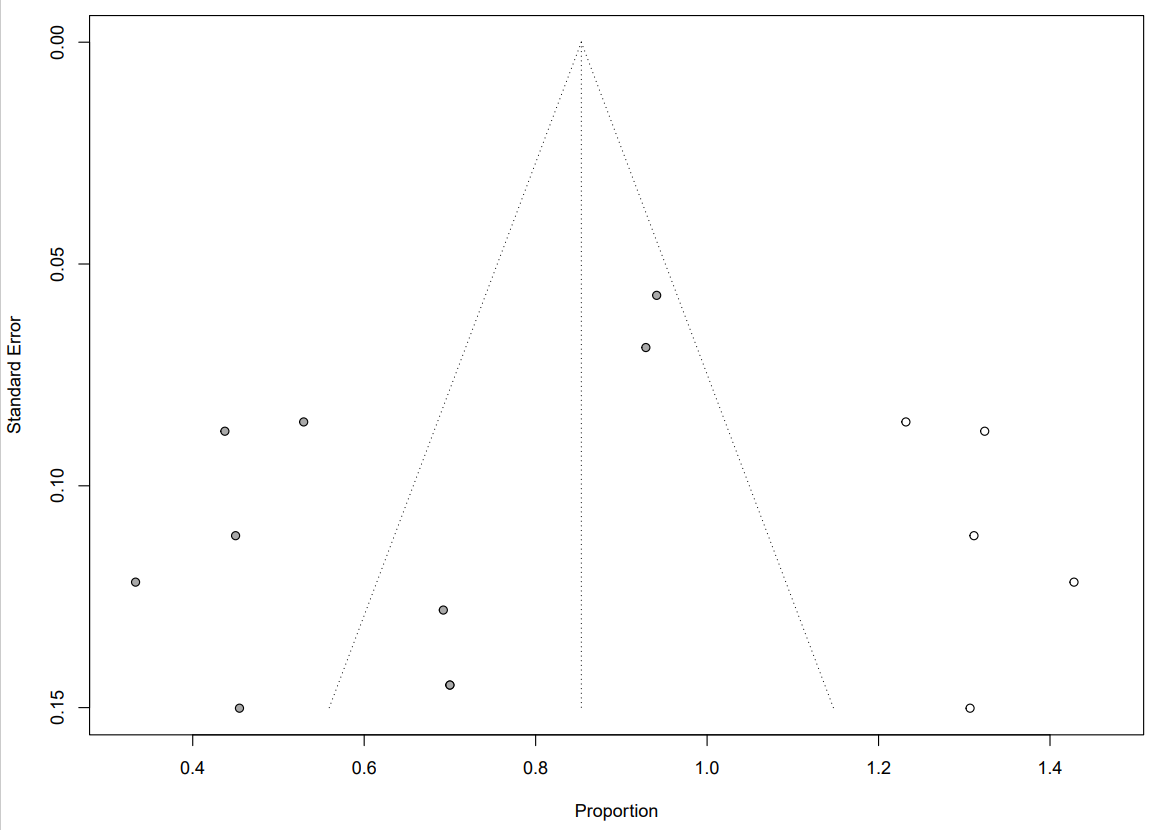


Figure S3. Funnel plot and trim-and-fill method examining the pooled DCR in ATC.
